# A retrospective, longitudinal study estimating the association between interdialytic weight gain and cardiovascular events and death in hemodialysis patients

**DOI:** 10.1186/s12882-015-0110-9

**Published:** 2015-07-22

**Authors:** Claudia Cabrera, Steven M. Brunelli, David Rosenbaum, Emmanuel Anum, Karthik Ramakrishnan, Donna E. Jensen, Nils-Olov Stålhammar, Bergur V. Stefánsson

**Affiliations:** AstraZeneca, R&D, SE-43183 Mölndal, Sweden; DaVita Clinical Research®, Minneapolis, MN USA; Ardelyx, Inc, Fremont, CA USA

**Keywords:** Blood pressure, Cardiovascular death, Chronic kidney disease, Fluid accumulation, Heart failure, Volume overload, Heart disease, Hemodialysis

## Abstract

**Background:**

Greater interdialytic weight gain (IDWG) is associated with risk of all-cause mortality and hospitalization. Dialysis patients are also at greater risk of cardiovascular (CV) events than patients without kidney disease. This retrospective study examined the potential association between IDWG and specific types of CV events.

**Methods:**

Data were obtained from United States Renal Data System claims and the electronic health records of Medicare patients who initiated hemodialysis between 01 January 2007 and 31 December 2008 at a large dialysis organization. Absolute IDWG was defined as predialysis weight minus postdialysis weight from the prior treatment, and relative IDWG was calculated as percentage of postdialysis weight with mean values for each, calculated over dialysis days 91 to 180. Patient outcomes were considered beginning on day 181, continuing until death, discontinuation of care, censoring, or study end (31 December 2009). Outcomes included all-cause mortality, CV mortality, hospitalization for nonfatal heart failure/volume overload, hospitalization for nonfatal myocardial infarction, MACE (a composite measure of nonfatal myocardial infarction, nonfatal ischemic stroke, or CV death), and MACE+ (events comprising MACE as well as arrhythmia, nonfatal hemorrhagic stroke, or hospitalization for heart failure). Associations between IDWG and outcomes over the exposure period were estimated using proportional hazards regression and adjusted for baseline characteristics.

**Results:**

39,256 patients qualified for analysis. In general, associations of relative IDWG with outcomes were more potent, consistent, and monotonic than those for absolute IDWG. Relative IDWG > 3.5 % body weight was independently associated with all outcomes studied: point estimates ranged from 1.18 (myocardial infarction) to 1.26 (CV mortality) and were consistent among patients with and without diabetes, and with and without baseline heart failure. Absolute IDWG > 3 kg was associated with outcomes other than myocardial infarction: point estimates ranged from 1.11 (MACE) to 1.20 (heart failure).

**Conclusions:**

Greater IDWG is associated with an increased risk of CV morbid events. Strategies that mitigate IDWG may improve CV health and survival among hemodialysis patients.

## Background

Patients with end-stage renal disease (ESRD) represent an important and increasingly prevalent portion of the medical patient population. There were 615,899 persons in the United States with ESRD in 2011, of which 430,273 were treated with dialysis [1], and the burden of concomitant illness is high in this patient population. Among ESRD patients treated with hemodialysis, the rate of hospitalization is 1.84/patient-year, with nearly one-third of these hospitalizations for cardiovascular (CV) causes [2]. Mitigating the rate of negative CV outcomes in dialysis patients has significant public health implications.

One plausible determination of poor CV outcomes in this population is interdialytic weight gain (IDWG). By virtue of implied limitations for renal excretion of endogenous salt and water and intermittent treatment schedules, thrice-weekly hemodialysis patients undergo repeated periods of fluid retention. Clinically, accumulated fluid is measured as IDWG (i.e., the change in weight from the end of one treatment until the beginning of the next). It has been demonstrated that greater IDWG is associated with a greater risk of all-cause mortality [3–5]. However, to date, there have been few studies directly examining the association between IDWG and CV morbid events. CV events are significant in their own right, occurring at a rate of approximately 510 admissions/1000 patient-years in hemodialysis patients and representing about 27 % of hospitalizations in this population [2], and also may serve as intermediary pathways linking IDWG to mortality.

This retrospective study examines the risk of CV events experienced by patients with ESRD undergoing hemodialysis. These analyses have been conducted to estimate the independent associative risks that exist between indices of interdialytic fluid accumulation and dialytic fluid removal with clinical outcomes, particularly, incident CV events and deaths. In addition, there is no consensus as to whether IDWG is best considered in absolute terms (i.e., kilograms of body weight change) or in relative terms (i.e., body weight change expressed as a proportion of body weight). To add clarity, we considered IDWG in both an absolute and relative manner and examined the comparative associations with outcomes.

## Methods

Retrospective data from patients in this study were extracted from the proprietary database of a large dialysis organization (LDO) in the United States. Eligible patients were those who initiated in-center hemodialysis at the LDO between 01 January 2007 and 31 December 2008 within 30 days of first dialysis. Because outcome data were abstracted from Medicare claims, analytic consideration was limited to patients with Medicare Part A primary insurance.

**Fig. 1:**
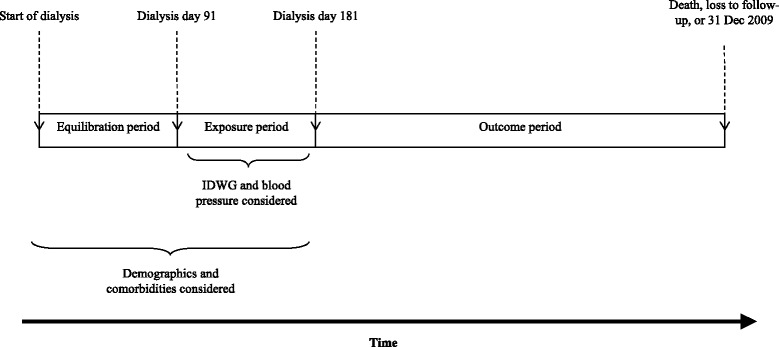
Study schema. Depicted are the time periods over which study data were considered. Abbreviations: IDWG, interdialytic weight gain

Upon dialysis initiation, patients undergo a period of adaptation during which target weight is identified, and dialysis treatments and medications are titrated before arriving at a quasi-steady state. To account for this, we did not consider IDWG over dialysis days 1 to 90 (the “equilibration period”) but instead over the 90-day period spanning from dialysis days 91 to 180 (the “exposure period”). Outcomes were considered beginning on dialysis day 181 and continuing until patients died, were censored for loss to follow-up (transfer of care, transplant, modality change, withdrawal from dialysis), or until 31 December 2009 (the “outcome period”). A schematic is provided in Fig. 1. Implicitly, patients who did not survive on hemodialysis until the start of the outcome period were excluded. This study design and patient population have been described previously [6].

Absolute IDWG was abstracted from patients’ electronic health records and was defined as predialysis weight from one treatment minus postdialysis weight from the prior treatment, which represents fluid accumulation between dialysis treatments (1 kg weight gain equals approximately 1 L fluid accumulated). Each patient’s mean IDWG value was then calculated over the exposure period. Absolute IDWG was considered as a continuous variable and also in a dichotomous sense; for the latter, a threshold of 3.0 kg was chosen based on empiric observations and prior literature [4]. Relative weight gain (relative IDWG) was calculated as a percentage of postdialysis weight. This measure described the amount of fluid accumulated between dialysis treatments as a function of the patient’s body size. Relative IDWG was considered as a continuous variable and also in a dichotomous sense; for the latter, a threshold of 3.5 % was chosen based on empiric observations and prior literature [7].

Covariates considered for the analysis included age, sex, race, vascular access type, etiology of ESRD, and prior renal transplant, as well as baseline history of diabetes, heart failure, myocardial infarction, atrial fibrillation, cerebrovascular disease (ischemic stroke, hemorrhagic stroke, or transient ischemic attack), and uncontrolled hypertension (mean predialysis blood pressure > 140/90 mm Hg or mean postdialysis blood pressure > 130/85 mm Hg during exposure period). Covariate data were abstracted from patients’ electronic health records and comorbidity data were supplemented from United States Renal Data System (USRDS) form 2728 data. Demographics were considered as of the start of the outcome period; comorbidities were considered based on all data available as of the start of the outcome period. IDWG and blood pressures, which were used to define uncontrolled hypertension, were considered over the exposure period.

The defined study outcomes were based on claims data from the Medicare Institutional Claims and Claims Detail Standard Analytical Files from the USRDS. The following outcomes were studied: all-cause mortality, CV mortality (defined as death attributed to myocardial infarction, atherosclerotic heart disease, cardiac arrhythmia, congestive heart failure, cardiomyopathy, cardiac arrest, valvular heart disease, pulmonary edema, cerebrovascular accident including intracranial hemorrhage, or ischemic brain damage/anoxic encephalopathy), hospitalization for nonfatal heart failure/volume overload, hospitalization for nonfatal myocardial infarction, and 2 composite endpoints for major adverse CV events [MACE (nonfatal myocardial infarction, nonfatal ischemic stroke, or CV death) and MACE+ (events comprising MACE as well as arrhythmia, nonfatal hemorrhagic stroke, or hospitalization for heart failure)].

Analyses were performed in parallel for absolute and relative IDWG. Baseline patient characteristics were described as means, SDs, counts, and proportions and compared across groups using t-tests and chi-squared tests. Associations with outcomes were estimated using Cox proportional hazards models. IDWG was considered as a restricted cubic spline and unadjusted associations with outcomes were estimated. Restricted cubic splines allow for flexible examination of associative patterns, with minimal assumptions regarding the shape of associations. Unadjusted and adjusted associations were estimated for dichotomous formulations of IDWG. The latter were adjusted for covariates listed above. For absolute IDWG, we fit additional models in which outcomes were also adjusted for body weight; relative IDWG was not adjusted for body weight because, upon body weight adjustment (i.e., when body weight is held analytically constant), relative IDWG is equivalent to absolute IDWG. Body weight was used in preference to body mass index due to missing height data. Finally, in considering CV mortality, we performed a sensitivity analysis using a competing risks model to account for the competing risk of non-CV mortality [8].

This study was performed retrospectively from de-identified electronic health records, and therefore deemed exempt by an institutional review board (Quorom).

## Results and discussion

**Fig. 2:**
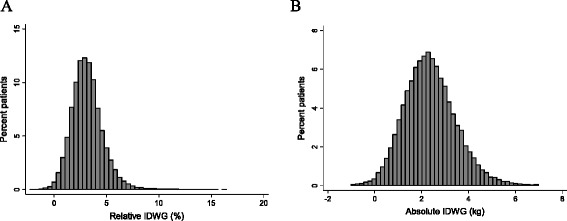
Distribution of interdialytic weight gain. Provided is the distribution of relative (panel **a**) and absolute IDWG (panel **b**) for the study cohort; IDWG was considered over dialysis days 91–180 as described in the text. Abbreviation: IDWG, interdialytic weight gain

Table 1 demonstrates baseline characteristics of the 39,256 patients qualifying for study. Overall, mean age was 62.2 years; 43.9 % were female, 46.0 % of patients were white, 31.7 % were black, and 14.7 % were Hispanic. Etiology of ESRD was diabetes in 47.1 %, hypertension in 30.4 %, glomerular disease in 7.2 %, and some other cause in 15.3 % of patients. At baseline, 68.2 % of patients had diabetes, 39.8 % had a history of heart failure, and 25.3 % had a history of myocardial infarction; 1.8 % of patients had received prior transplants and were initiating hemodialysis in the setting of a failed transplant. The distributions of relative and absolute IDWG are shown in Fig. 2. Mean relative IDWG was 3.1 %; the distribution was noticeably right skewed. Mean absolute IDWG was 2.4 kg; the distribution was more symmetrical than relative IDWG distribution, but still somewhat right skewed.

**Table 1:** Baseline characteristics of the cohort overall and stratified on relative and absolute interdialytic weight gain

Variable^a^	Overall N = 39,256	Relative IDWG			Absolute IDWG		
		≤3.5 % n = 24,726	>3.5 % n = 14,530	p-value	≤3.0 kg n = 28,942	>3.0 kg n = 10,314	p-value
Age, year	62.2 ± 15.3	63.0 ± 15.0	60.7 ± 15.6	<0.001	63.7 ± (15.3	57.9 ± 14.2	<0.001
Female sex	17,238 (43.9)	11,462 (46.4)	5776 (39.8 %)	<0.001	14,180 (49.0)	3058 (29.7)	<0.001
Race				<0.001			<0.001
White	18,055 (46.0)	11,975 (48.5)	6080 (41.9)		13,358 (46.2)	4697 (45.6)	
Black	12,437 (31.7)	7951 (32.2)	4486 (30.9)		9008 (31.1)	3429 (33.3)	
Hispanic	5777 (14.7)	3153 (12.8)	2624 (18.1)		4279 (14.8)	1498 (14.5)	
Asian	1260 (3.2)	635 (2.6)	625 (4.3)		1044 (3.6)	216 (2.1)	
Other	984 (2.5)	586 (2.4)	398 (2.7)		742 (2.6)	242 (2.4)	
ESRD etiology				<0.001			<0.001
Diabetes	18,500 (47.1)	11,068 (44.8)	7432 (51.2)		12,649 (43.7)	5851 (56.7)	
Hypertension	11,942 (30.4)	7834 (31.7)	4108 (28.3)		9361 (32.3)	2581 (25.0)	
Glomerular disease	2825 (7.2)	1927 (7.8)	898 (6.2)		2190 (7.6)	635 (6.2)	
Other	5989 (15.3)	3897 (15.8)	2082 (14.4)		4742 (16.4)	1247 (12.1)	
Prior renal txp	697 (1.8)	366 (1.5)	331 (2.3)	<0.001	507 (1.8)	190 (1.8)	0.55
Baseline diabetes^b^	26,768 (68.2)	16,337 (66.1)	10,431 (71.8)	<0.001	18,765 (64.8)	8003 (77.6)	<0.001
Baseline CHF^b^	15,623 (39.8)	9340 (37.8)	6283 (43.2)	<0.001	11,008 (38.0)	4615 (44.8)	<0.001
Baseline MI^b^	9920 (25.3)	6238 (25.2)	3682 (25.3)	0.81	7274 (25.1)	2646 (25.7)	0.30
Baseline AF^b^	2310 (5.9)	1433 (5.8)	877 (6.0)	0.33	1720 (5.9)	590 (5.7)	0.41
Baseline CVD^c^	3404 (8.7)	2129 (8.6)	1275 (8.8)	0.58	2560 (8.9)	844 (8.2)	0.04
Uncontrolled hypertension^d^	37,283 (95.0)	23,492 (95.0)	13,791 (94.9)	0.68	27,464 (94.9)	9819 (95.2)	0.22
Vascular access				<0.001			<0.001
AVF	10,787 (27.7)	6826 (27.6)	4052 (27.9)		7728 (26.7)	3150 (30.6)	
AVG	3782 (9.6)	2269 (9.2)	1513 (10.4)		2776 (9.6)	1006 (9.8)	
CVC	24,573 (62.6)	15,616 (63.2)	8957 (61.7)		18,420 (63.7)	6153 (59.7)	
Postdialysis weight, kg	77.0 (34.7)	80.3 (41.2)	71.4 (17.8)	<0.001	73.3 (37.2)	87.4 (23.5)	<0.001

*Abbreviations: AF* atrial fibrillation, CHF congestive heart failure, *CVD* cerebrovascular disease, *AVF* arteriovenous fistula, *AVG* arteriovenous graft, *CVC* central venous catheter, *ESRD* end-stage renal disease, *IDWG* interdialytic weight gain, *MI* myocardial infarction, *txp* transplant

^a^Values expressed as mean ± SD or n (%)

^b^Defined based on CMS Medical Evidence Form 2728 data, claims (1 inpatient or 2 outpatient), or LDO EHR records prior to dialysis day 180

^c^Defined based on CMS Medical Evidence Form 2728, claims (1 inpatient or 2 outpatient), LDO EHR record prior to dialysis day 180. Includes ischemic stroke, hemorrhagic stroke, and TIA

^d^Defined as mean predialysis blood pressure > 140/90 mm Hg or postdialysis blood pressure > 130/85 mm Hg during the outcome period (dialysis days 91–180)

Incidence rates for events of interest during the outcome period are provided in Table 2. Hospitalizations for nonfatal myocardial infarction occurred at a rate of 59.5 events/1000 patient-years, hospitalizations for heart failure/volume overload at a rate of 243 events/1000 patient-years, MACE at a rate of 108 events/1000 patient-years, MACE+ at a rate of 324 events/1000 patient-years, CV mortality at a rate of 55.8 deaths/1000 patient-years, and all-cause mortality at a rate of 142 deaths/1000 patient-years.

**Table 2:** Event count and incidence rates for outcomes of interest during the outcome period

Outcomes	Number of patients affected	Cumulative time at-risk, 1000 patient-years	Median time at-risk, days (p25, p50)	Incidence rate to first event, 1000 patient-years (95 % CI)
MI	2378	40.0	386 (221, 588)	59.5 (57.1–61.9)
HF	8802	36.2	340 (184, 543)	243 (238–248)
MACE^a^	4314	40.1	387 (221, 589)	108 (105–111)
MACE+^b^	11,488	35.4	330 (174, 533)	324 (381–330)
CV mortality^c^	2294	41.1	398 (232, 602)	55.8 (53.6–58.2)
All-cause mortality	5818	41.1	398 (232, 602)	142 (138–145)

*Abbreviations: p25* twenty-fifth quartile, *p50* fiftieth quartile, *CI* confidence interval, *CV* cardiovascular, *HF* heart failure/volume overload, *MACE* major adverse cardiovascular event, *MI* myocardial infarction

^a^Defined as nonfatal myocardial infarction, nonfatal ischemic stroke, or CV death

^b^Defined as events comprising MACE as well as arrhythmia, nonfatal hemorrhagic stroke, or hospitalization for heart failure

^c^Defined as death attributed to MI, atherosclerotic heart disease, cardiac arrhythmia, congestive HF, cardiomyopathy, cardiac arrest, valvular heart disease, pulmonary edema, or cerebrovascular accident including intracranial hemorrhage or ischemic brain damage/anoxic encephalopathy

**Fig. 3:**
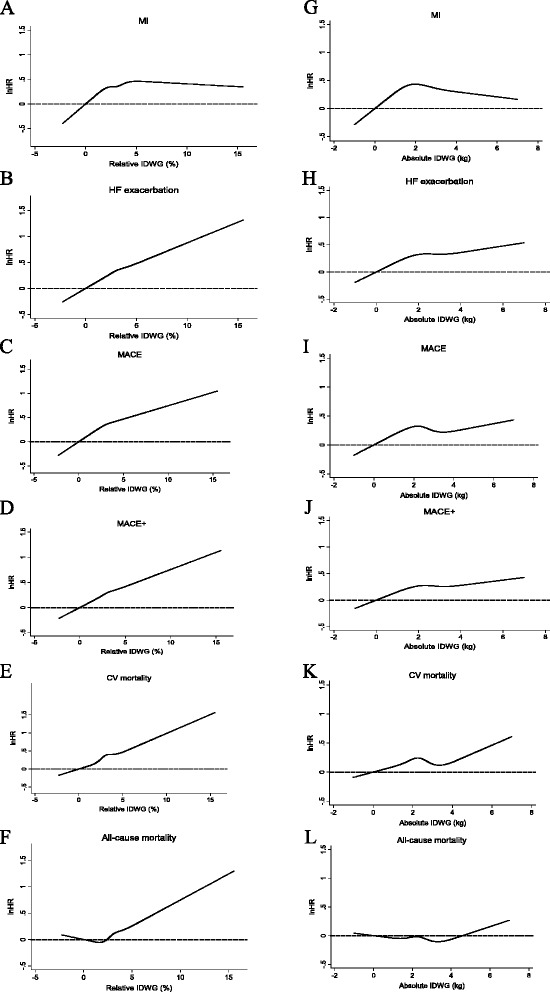
Unadjusted associations of relative (panels **a**-**f**) and absolute (panels **g**-**l**) interdialytic weight gain with outcomes of interest. In each analysis, IDWG was modeled as a restricted cubic spline. Displayed are point estimates (line) and 95 % CI (shaded areas). Abbreviations: CI, confidence interval; CV, cardiovascular; HF, heart failure/volume overload; HR, hazard ratio; IDWG, interdialytic weight gain; MACE, major adverse cardiovascular event; MI, myocardial infarction

Unadjusted associations of absolute and relative IDWG with each outcome of interest are shown in Fig. 3. In general, associations with an outcome were more potent for relative versus absolute IDWG. For most outcomes, associations with relative IDWG were relatively monotonic, whereas the association with absolute IDWG was less consistent and demonstrated threshold effects. For myocardial infarction, a threshold effect was observed with relative IDWG as well; nonetheless, the association was more potent for relative versus absolute IDWG.

Considered dichotomously, compared to patients with low relative and absolute IDWG, respectively, patients with high relative (>3.5 %) and absolute IDWG (>3 kg), respectively, were on average younger and less likely to dialyze via a catheter, but were more likely to have baseline diabetes or heart failure. These and other comparisons are presented in Table 1. Associations were synchronous except that high relative IDWG was associated with lower body weight whereas high absolute IDWG was associated with higher body weight.

Table 3 presents unadjusted and adjusted associations between absolute and relative IDWG and endpoints during the outcome period. On an unadjusted basis, higher relative IDWG was associated with greater risk of each outcome studied. Upon multivariable adjustment, estimates were slightly potentiated but qualitatively similar. Measures of associations ranged from 14 % greater risk (myocardial infarction) to 26 % greater risk (CV mortality).

**Table 3:** Adjusted and unadjusted associations between interdialytic weight gain and outcomes of interest

	Relative IDWG		Absolute IDWG		
Outcomes	Unadjusted HR (95 % CI)	Adjusted^d^ HR (95 % CI)	Unadjusted HR (95 % CI)	Adjusted^d^ HR (95 % CI)	Adjusted^d^ + postdialysis weight HR (95 % CI)
	>3.5 % (vs ref ≤ 3.5 %)	>3.5 % (vs ref ≤ 3.5 %)	>3 kg (vs ref ≤ 3 kg)	>3 kg (vs ref ≤ 3 kg)	>3 kg (vs ref ≤ 3 kg)
MI	1.14 (1.05–1.24)	1.18 (1.08–1.28)	0.94 (0.86–1.03)	1.02 (0.93–1.12)	1.07 (0.97–1.18)
HF	1.19 (1.14–1.24)	1.20 (1.15–1.26)	1.08 (1.03–1.13)	1.14 (1.09–1.20)	1.20 (1.14–1.26)
MACE^a^	1.19 (1.12–1.27)	1.21 (1.14–1.29)	0.98 (0.92–1.05)	1.04 (0.97–1.12)	1.11 (1.03–1.19)
MACE+^b^	1.18 (1.14–1.23)	1.22 (1.17–1.26)	1.05 (1.01–1.10)	1.14 (1.09–1.19)	1.19 (1.14–1.25)
CV mortality^c^	1.23 (1.14–1.34)	1.23 (1.13–1.34)	1.00 (0.91–1.09)	1.04 (0.94–1.15)	1.12 (1.02–1.24)
All-cause mortality	1.22 (1.16–1.29)	1.26 (1.20–1.33)	0.97 (0.92–1.03)	1.07 (1.00–1.13)	1.17 (1.10–1.24)

*Abbreviations: CI* confidence interval, *CV* cardiovascular, *MI* myocardial infarction, *HF* heart failure/volume overload, *HR* hazard ratio, *IDWG* interdialytic weight gain, *MACE* major adverse cardiovascular event, *ref* reference

^a^Defined as nonfatal myocardial infarction, nonfatal ischemic stroke, or CV death

^b^Defined as events comprising MACE as well as arrhythmia, nonfatal hemorrhagic stroke, or hospitalization for heart failure

^c^Defined as death attributed to MI, atherosclerotic heart disease, cardiac arrhythmia, congestive HF, cardiomyopathy, cardiac arrest, valvular heart disease, pulmonary edema, or cerebrovascular accident including intracranial hemorrhage or ischemic brain damage/anoxic encephalopathy

^d^HRs were adjusted for age, race, sex, etiology of end-stage renal disease, prior renal transplant, access type, and baseline diabetes, uncontrolled hypertension, HF, MI, atrial fibrillation, and ischemic/hemorrhagic stroke or transient ischemic attack

On an unadjusted basis, higher absolute IDWG was associated with a greater risk of hospitalization for heart failure/volume overload and MACE+, but not other outcomes. Upon multivariable adjustment, estimates were mildly potentiated; significant adjusted associations were observed for all-cause mortality (7 % greater risk), heart failure/volume overload (14 % greater risk), and MACE+ (14 % greater risk). Upon further adjustment for body weight, greater absolute IDWG was associated with all outcomes considered except for heart failure. Measures of association ranged from 11 % (MACE) to 20 % (heart failure/volume overload).

**Fig. 4:**
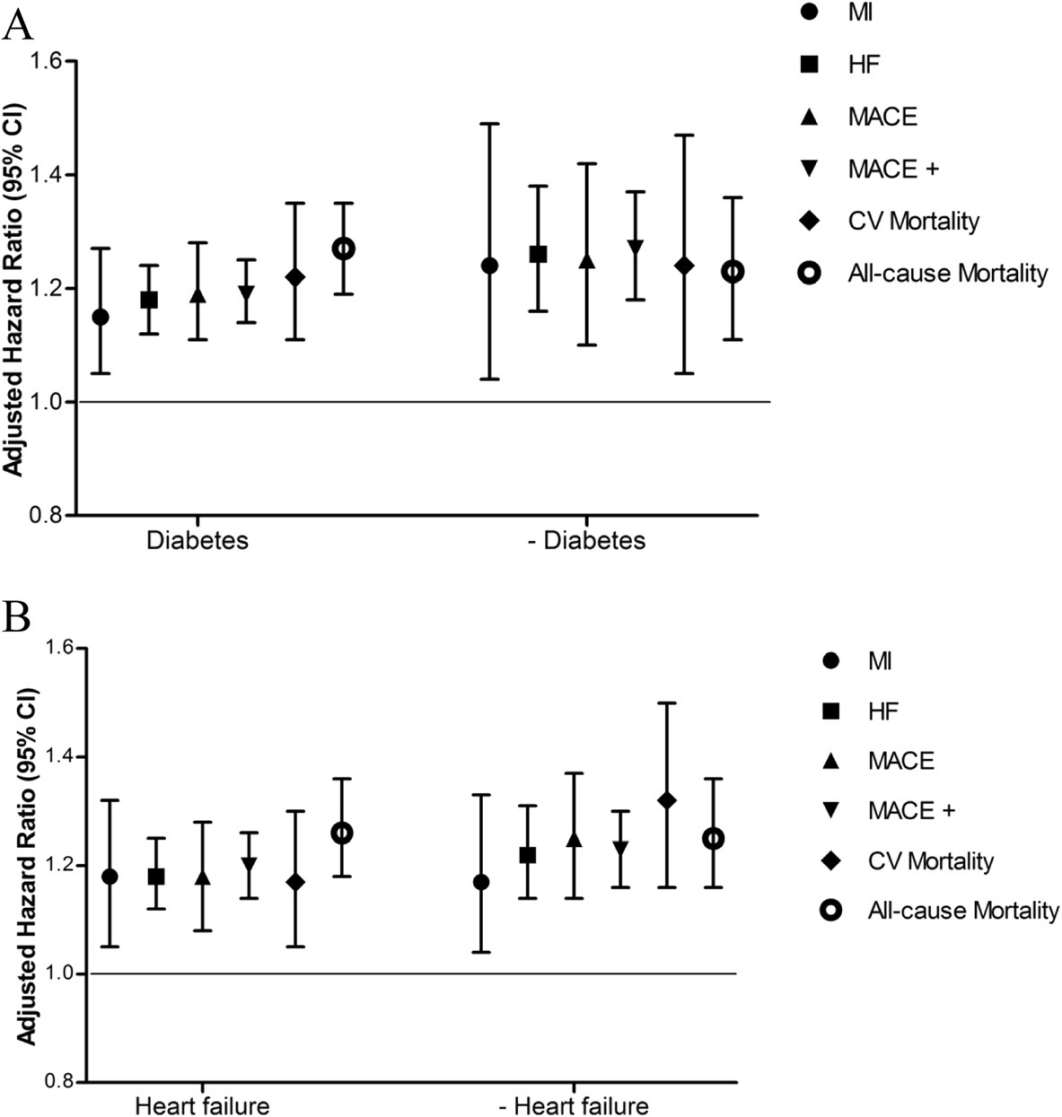
Adjusted associations of relative interdialytic weight gain with outcomes of interest in subgroups of patients with and without baseline diabetes (panel **a**) and heart failure (panel **b**). HRs are shown with 95 % CIs. HRs were adjusted for age, race, sex, etiology of end-stage renal disease, prior renal transplant, access type, and baseline diabetes, uncontrolled hypertension, HF, MI, atrial fibrillation, and ischemic/hemorrhagic stroke or transient ischemic attack. Abbreviations: CI, confidence interval; CV, cardiovascular; HF, heart failure/volume overload; HR, hazard ratio; MACE, major adverse cardiovascular event; MI, myocardial infarction

To determine whether risk of poor outcomes might be modified by underlying diabetes or baseline heart failure, we conducted restriction subgroup analyses (Fig. 4). Results demonstrate that associations of high relative IDWG with outcomes were similar in diabetic and nondiabetic patients and in patients with and without baseline heart failure.

Finally, a sensitivity analysis was conducted in which the association between high relative IDWG and CV mortality was analyzed under a competing risks framework. The association was not meaningfully different from that observed in the primary analysis: hazard ratio (95 % CI) for relative IDWG > 3.5 % was 1.26 (1.20–1.33).

CV-related morbidity and mortality are greater in ESRD patients compared to the general population [9]. This is probably related to the vast constellation of underlying conditions that contribute to the deterioration of the circulatory system, such as chronic hypervolemia, high blood pressure, and periodic episodes of intravascular hypovolemia with attendant tissue hypoxia, all superimposed on a background of vascular disease, diabetes, and autonomic nervous system dysfunction. These data suggest that IDWG may contribute independently and substantively to CV burden.

For the most part, dialysis patients are completely reliant on the dialysis procedure for fluid removal. There is sound biologic basis as to why greater IDWG may be harmful. Acutely, greater volumes of retained fluid increase cardiac filling pressures and predispose patients to left ventricular strain, pulmonary edema, and excessive blood pressure. In addition, because dialysis treatment time is essentially fixed for most US patients, greater IDWG implies a greater rate of fluid removal during dialysis (i.e., ultrafiltration), which is associated with hemodynamic instability and death [4, 10–14]. Chronic or repeated episodes of fluid retention contribute to maladaptive changes in cardiac structure, such as left ventricular hypertrophy and fibrosis, which may distort electrical conduction pathways and promote ventricular tachyarrhythmia and sudden cardiac death. Moreover, data demonstrate that asymptomatic pulmonary congestion and overhydration on bioimpedance are potently associated with increased risk of death in otherwise stable hemodialysis patients [15, 16]. Hur et al. found significant improvements of left ventricular hypertrophy, blood pressure, and pulse wave velocity targeting normohydration amid the interdialytic period instead of the end of the dialysis session with the help of bioimpedance, implying that restricting fluid exposure reduces CV complications [17]. There are several plausible mechanisms behind the findings, including stunning, strain of IDWG itself, or dry weight that is greater than optimal, as these patients are difficult to bring down to normohydration. Further, it is possible that IDWG necessitates rapid ultrafiltration since the duration of dialysis is fixed from a practical standpoint. However, the distinction between these 2 circumstances is not possible with the current study design, but shows only that minimizing IDWG is potentially important. Earlier studies have demonstrated that ESRD patients experiencing large IDWG have an increased risk of death compared to those with lesser weight gain between dialysis sessions [4, 13].

The results presented here are consistent and extend prior findings by demonstrating potent associations between IDWG and CV morbid events and CV mortality [3, 18]. In building upon these prior data, the present findings are noteworthy because they provide a physiologic and clinical link between fluid accumulation and mortality, demonstrate profound clinical and economic implications, and imply that mitigating IDWG could improve health and survival among ESRD patients. Unlike past studies that considered only composite endpoints, our robust sample size enabled us to assess associations with individual outcomes of interest. Moreover, it afforded greater statistical power to detect associations between smaller increments in IDWG and outcomes than have been previously reported.

There are 2 ways of expressing IDWG for clinical and research purposes: absolute IDWG and relative IDWG, the latter expressed as a percentage of body weight. There is no clear consensus in the literature as to which of these metrics is more clinically relevant. Our data indicate that relative IDWG is more potently, consistently, and monotonically associated with the outcomes studied than is absolute IDWG, and therefore may be the more relevant parameter. If so, this would suggest that small patients may be particularly vulnerable to the effects of high IDWG by virtue of having less ability to “store” excess fluid between dialysis sessions. However, inference in this regard should be undertaken cautiously. High relative IDWG was associated with smaller body size whereas high absolute IDWG was associated with larger body size. Considering the known association between body size and clinical outcomes [19], this distinction could explain the discrepancies observed. Of note in this regard is that the associations between absolute IDWG and outcomes were potentiated upon adjustment for body weight. Analogous adjustment for relative IDWG could not be undertaken because—when body weight is held analytically constant as in the case of statistical adjustment—absolute and relative IDWG converge to the same construct. Further research is needed to elaborate the contributions of relative and absolute IDWG, including dilutional studies demonstrating distribution of accumulated fluid within relevant body compartments. Until such is known, the most conservative interpretation of these findings would be to consider the weight-adjusted outcomes for absolute IDWG: that greater IDWG is associated with heart failure exacerbation, MACE, MACE+, CV mortality, and all-cause mortality but not with myocardial infarction.

Study limitations include the retrospective design of this analysis, which can lead to confounding. Specific to this study, we lacked data on residual urine output. This could potentially have confounded findings if, and to the degree that, residual urine output impacts outcomes independently of effects mediated through IDWG. There may have been residual confounding due to comorbid conditions not considered, or the severity of those that were. It is noteworthy that in the study by Lee et al., high relative IDWG was associated with an increased risk of MACE, even after adjustment for urine production and nutritional status [18]. Another confounding factor to acknowledge is that patients may have been nonadherent to dialysis, leading to larger IDWG between sessions, thereby inflating the measurement of IDWG. Again, this could confound findings to the degree that poor dialysis attendance impacted outcomes independent of intermediary effects on IDWG. The duration of follow-up in this study was relatively short (potential follow-up time ranged from 1–3 years based on the date of accrual). However, normative data demonstrate that median survival time for hemodialysis patients in the United States is only 3 years, and thus our time horizon is relevant [20].

Finally, of note this study considered IDWG. This is a separate, though related concept to chronic fluid accumulation (eg, as measured by bioimpedance, online hematocrit monitoring, or soluble biomarkers) [21, 22]. Additional studies are needed to better understand how cyclical fluid changes represented by IDWG and chronic volume expansion interact with respect to clinical outcomes.

## Conclusions

In summary, the current analysis has demonstrated potent and independent associations between greater IDWG and risk of CV events, particularly hospitalization for heart failure/volume overload and mortality. Observational research and clinical trials are needed to evaluate if current and upcoming strategies and therapies that mitigate IDWG may improve CV health and survival among ESRD patients.

## Abbreviations

CI: Confidence interval
CV: Cardiovascular
ESRD: End-stage renal disease
HF: Heart failure
HR: Hazard ratio
IDWG: Interdialytic weight gain
MACE: Major adverse cardiovascular event
MI: Myocardial infarction
ref: Reference
CMS: Centers for Medicare and Medicaid Services
EHR: Electronic health record
TIA: Transient ischemic attack
